# Effect of antenatal milk expression education on lactation outcomes in birthing people with pre-pregnancy body mass index ≥25: protocol for a randomized, controlled trial

**DOI:** 10.1186/s13006-023-00552-6

**Published:** 2023-03-16

**Authors:** Jill R. Demirci, Melissa Glasser, Debra L. Bogen, Susan M. Sereika, Dianxu Ren, Kristin Ray, Lisa M. Bodnar, Therese A. O’Sullivan, Katherine Himes

**Affiliations:** 1 Department of Health Promotion & Development, University of Pittsburgh School of Nursing, Pittsburgh, PA USA; 2 Allegheny County Health Department, Pittsburgh, PA USA; 3 Department of Health & Community Systems, University of Pittsburgh School of Nursing, Pittsburgh, PA USA; 4 Department of Pediatrics, University of Pittsburgh School of Medicine, Pittsburgh, PA USA; 5 UPMC Children’s Community Pediatrics, Pittsburgh, PA USA; 6 Department of Epidemiology, University of Pittsburgh School of Public Health, Pittsburgh, PA USA; 7 School of Medical and Health Sciences, Edith Cowan University, Joondalup, WA Australia; 8 Department of Obstetrics, Gynecology, and Reproductive Sciences, Division of Maternal-Fetal Medicine, University of Pittsburgh School of Medicine, Pittsburgh, PA USA; 9 UPMC Magee-Womens Hospital, Pittsburgh, PA USA

**Keywords:** Antenatal milk expression, Antenatal colostrum harvesting, Telelactation, Breastfeeding, Breast milk expression, Breast milk collection, Overweight, Obesity, Body mass index, Pregnancy

## Abstract

**Background:**

Birthing people with pre-pregnancy body mass indices (BMIs) ≥ 25 kg/m^2^, particularly those without prior breastfeeding experience, are at increased risk for suboptimal lactation outcomes. Antenatal milk expression (AME) may be one way to counteract the negative effects of early infant formula supplementation common in this population.

**Methods:**

This ongoing, randomized controlled trial in the United States evaluates the efficacy of a telelactation-delivered AME education intervention versus an attention control condition on lactation outcomes to 1 year postpartum among 280 nulliparous-to-primiparous, non-diabetic birthing people with pre-pregnancy BMI ≥ 25 kg/m^2^. The assigned study treatment is delivered via four weekly online video consultations between gestational weeks 37–40. Participants assigned to AME meet with study personnel and a lactation consultant to learn and practice AME. Instructions are provided for home practice of AME between study visits. Control group participants view videos on infant care/development at study visits. Participants complete emailed surveys at enrollment (34^0/7^–36^6/7^ gestational weeks) and 2 weeks, 6 weeks, 12 weeks, 6 months, and 12 months postpartum. Surveys assess lactation and infant feeding practices; breastfeeding self-efficacy, attitudes, and satisfaction; perception of insufficient milk; onset of lactogenesis-II; lactation support and problems; and reasons for breastfeeding cessation. Surveys also assess factors associated with lactation outcomes, including demographic characteristics, health problems, birth trauma, racial discrimination, and weight stigma. Health information and infant feeding data are abstracted from the pregnancy and birth center electronic health record. Milk samples are collected from the intervention group at each study visit and from both groups at each postpartum follow-up for future analyses. Qualitative interviews are conducted at 6 weeks postpartum to understand AME experiences. Primary outcomes of interest are breastfeeding exclusivity and breastfeeding self-efficacy scores at 2 weeks postpartum. Outcomes will be examined longitudinally with generalized linear mixed-effects modeling.

**Discussion:**

This is the first adequately powered trial evaluating the effectiveness of AME among U.S. birthing people and within a non-diabetic population with pre-pregnancy BMI ≥ 25 kg/m^2^. This study will also provide the first evidence of acceptability and effectiveness of telelactation-delivered AME.

**Trial registration:**

ClinicalTrials.gov: NCT04258709.

## Background

Birthing people with pre-pregnancy overweight or obesity (body mass index, BMI ≥ 25 kg/m^2^) initiate and continue direct chest/breastfeeding and/or provision of their own milk (hereafter, collectively referred to as “breastfeeding”) at rates significantly below the general population [1]. Compared to birthing people with BMI < 25 kg/m^2^ prior to pregnancy, those with pre-pregnancy BMIs ≥25 kg/m^2^ have up to a four-fold increased risk of breastfeeding non-initiation [2] and up to a three-fold increased risk of non-exclusive or no breastfeeding at 2, 4, and 6 months postpartum [3–5].

The reasons for suboptimal breastfeeding in this population are multifactorial and include physiological, psychological, mechanical, and support barriers. For example, certain metabolic conditions are more common among individuals with high pre-pregnancy BMI (e.g., polycystic ovarian syndrome, metabolic syndrome), which can negatively impact glandular breast tissue development and hormonal signaling necessary for milk production [6, 7]. Birthing people with overweight/obesity are also at higher risk for birth complications (e.g., prolonged labor, cesarean sections), infant morbidity (e.g., prematurity, hypoglycemia), and pregnancy morbidity (e.g., preeclampsia, gestational hypertension), which may contribute to early parent-infant separation, delayed onset of copious milk production (lactogenesis II), and early formula use [6, 8]. Other barriers to breastfeeding among birthing people with higher pre-pregnancy BMI may include lack of representation in breastfeeding promotion and education materials, body image concerns and discomfort with breast exposure during lactation, difficulty with breastfeeding positioning with larger breasts, and implicit bias from healthcare professionals who provide lactation support [6, 8].

Supporting individuals with overweight/obesity to breastfeed has significant individual and public health implications. The number of birthing people and their offspring with overweight/obesity and related morbidities is growing in the United States (U.S.) [9–11]. Breastfeeding can substantially reduce the development of many of these morbidities (e.g., maternal and childhood diabetes, maternal cardiovascular disease) [12–14].

Few lactation support resources or interventions exist to address the combination of issues birthing people with overweight or obesity may encounter in establishing breastfeeding. Antenatal milk expression (AME)—a practice growing in global popularity [15], may offer one such solution. AME entails hand expression of colostrum in pregnancy, usually commencing between 36 and 37 weeks of gestation. Expressed milk may be collected, frozen, and used for supplementation of direct breastfeeding after birth if needed [15].

Among birthing people with diabetes, whose infants are at risk for early formula use due to postpartum hypoglycemia, there is evidence that AME may increase prenatal and postnatal breastfeeding confidence in some cases [16–18] and reduce birth hospital formula supplementation [19–21]. The largest trial to date of AME, the DAME Trial, provided evidence of AME’s safety. The DAME Trial involved 635 women with gestational or preexisting diabetes at low risk for other perinatal complications, 319 of whom were randomized to practice AME twice daily beginning at 36 weeks of pregnancy. The study team found that AME did not influence infant gestational age or neonatal intensive care unit (NICU) admissions, nor was it associated with uterine hyperstimulation or fetal compromise [19]. In an online survey conducted with 688 mothers in the UK regarding their perceptions of AME, 81% were amenable to trying AME. AME was considered especially beneficial in the case of maternal or infant medical problems, with positive opinions about AME more prevalent among women with increasing BMI (30% of women with BMI < 25 kg/m^2^ endorsed “AME is good idea” versus 32 and 47% of women with BMI 25–29.9 kg/m^2^ and BMI ≥ 30 kg/m^2^, respectively) [22].

For first-time birthing people and those with overweight or obesity in particular, AME has the potential to influence lactation outcomes through several mechanisms. First, AME can build confidence with chest/breast exposure and milk expression mechanics through scaffolded practice, occurring in a low stakes prenatal environment [17, 18] and prior to what may be a complicated birth and recovery [23, 24]. Second, if parents engaging in AME collect and save their milk prior to birth, it can be used for early supplementation of direct breastfeeding when indicated or advised, in lieu of infant formula. Supplementation with infant formula in the first days postpartum among women intending to exclusively breastfeed is associated with increased risk of non-exclusive breastfeeding and breastfeeding cessation by 1–2 months postpartum [25]. Third, the period immediately proximal to birth is considered a critical window during which milk expression/removal is hypothesized to favorably influence short- and long- term milk production, possibly through up-regulation of prolactin receptors in breast tissue [26–28]. This critical window may extend to the antenatal period, with some qualitative accounts of individuals attributing their abundant postpartum milk volumes to AME [21]. This may be particularly salient for nulliparous-to-primiparous individuals (i.e., first-time birthing people) with overweight or obesity, who are at higher risk for insufficient milk production and delayed lactogenesis II [29–31].

The feasibility and efficacy of AME as a lactation support intervention for individuals with overweight or obesity without diabetes has not been previously investigated. The purpose of the PREPARE (PRenatal Video-Based Education and Post-PARtum Effects) Trial is to evaluate the efficacy of a telelactation-delivered AME intervention on short- and long- term lactation outcomes to 1 year postpartum among a sample of nulliparous-to-primiparous, non-diabetic birthing people with pre-pregnancy BMIs ≥25 kg/m^2^. A secondary aim is to explore participants’ experiences with and perceptions of AME.

## Methods/design

### Design

The PREPARE Trial is an ongoing two-arm, parallel group, superiority randomized controlled trial with 1:1 allocation ratio based in the U.S. Participants are randomly assigned to either: 1) AME instruction/education delivered by remote, live International Board-Certified Lactation Consultants (IBCLCs), with instructions to engage in AME self-practice 1–2 times per day; or 2) an attention control condition receiving infant care education unrelated to infant feeding via short videos. In both groups, participants receive their assigned intervention during weekly remote video visits (via Zoom) with the study team between 37^0/7^ and 40^6/7^ weeks gestation. Lactation outcomes and experiences with the assigned intervention are assessed via electronic surveys, phone interviews, and review of electronic health record (EHR) data. Data are collected at enrollment, weekly prenatal study visits, during the postpartum birth center admittance, and at 2 weeks, 6 weeks, 12 weeks, 6 months, and 12 months postpartum.

Participant recruitment for the PREPARE Trial began in September 2020. We expect to complete recruitment in 2024 and data collection in 2025.

### Hypotheses and outcome variables

#### Primary hypotheses and outcomes

We hypothesize that compared to the attention control group, participants receiving the AME intervention will have higher breastfeeding self-efficacy and higher rates of exclusive breastfeeding within the first two postpartum weeks. To examine these hypotheses, we are measuring the following primary outcomes:

- exclusive provision of participant’s own milk at 2 weeks postpartum (self-report)
- scores on the Breastfeeding Self-Efficacy Scale-Short Form (SF) [32] at 2 weeks postpartum

#### Exploratory aims and outcomes

We will examine the potential impact of AME on other short- and long-term lactation outcomes, including:

- breastfeeding initiation and duration: any provision of participant’s own milk at each postpartum assessment point: postpartum birth center admittance (EHR); 2 weeks, 6 weeks, 12 weeks, 6 months, and 12 months postpartum (self-report)
- breastfeeding exclusivity: provision of only participant’s own milk and proportional range of participant’s own milk versus other foods/infant formula during the postpartum birth center admittance (EHR), from birth to 2 weeks, and at 2 weeks, 6 weeks, 12 weeks, and 6 months postpartum (self-report)
- breastfeeding self-efficacy: scores on the Breastfeeding Self-Efficacy Scale-SF at 6 and 12 weeks postpartum
- perceived milk supply: Score on Perceived Infant Breastfeeding Satiety Subscale (PIBSS) of the H & H Lactation Scale [33] measuring perceived infant satisfaction with breast milk received; endorsement of insufficient milk supply via investigator-created item at 2, 6, and 12 weeks postpartum
- onset of lactogenesis II: self-report recall in post-birth days, assessed at 2 weeks postpartum
- experiences and perceptions of AME: qualitative interview with subset of participants at 6 weeks postpartum or upon notification of cessation of any feeds of participant’s own milk, if prior to 6 weeks

See Table 1 for a complete description of outcome variables and their measurement.

**Table 1:** Primary and secondary outcome variables and measurement

Variable	Measurement	Source	Assessment timing
Primary Outcome Variables			
Breastfeeding exclusivity	Current provision of only participant’s own milk to infant	Survey item adapted from Infant Feeding Practices Survey-II (IFPS-II) [34]	PP wk. 2
Breastfeeding self-efficacy	Sum scale score on instrument assessing extent of agreement with statements about breastfeeding confidence on Likert scale	Prenatal and Postpartum Breastfeeding Self-Efficacy Scale-SF [32]^a^ (14 items)	Enrollment; PP wk. 2
Secondary Outcome Variables (*indicates an outcome that is also a mediating variable)			
Breastfeeding exclusivity	Estimated proportion of participant’s own milk feeds in: a) past week; and b) since birth (2 weeks pp); proportion of participant’s own milk feeds during birth center admittance; recall of infant age (days/weeks) to first formula use	Survey items adapted from Infant Feeding Practices Survey-II (IFPS-II) [34] and National Immunization Survey (NIS) [35]; electronic health record (EHR)	Birth center admittance; PP wks 2, 6, 12; 6 & 12 mos
Breastfeeding duration	Current provision and birth center provision of participant’s own milk; recall of infant age when feeds of participant’s own milk ceased	Survey items adapted from NIS; birth center data abstracted from EHR	Birth center admittance; PP wks 2, 6, 12; 6 & 12 mos
Breastfeeding self-efficacy	Sum scale score on instrument assessing extent of agreement with statements about breastfeeding confidence on Likert scale	Prenatal and Postpartum Breastfeeding Self-Efficacy Scale-SF [32]^a^ (14 items)	Enrollment; PP wks, 6, 12
*Onset of lactogenesis II	Recall of lactogenesis II in post-birth days with item “When did your milk come in after your baby was born (i.e., when did you notice a big increase in the amount of milk)?” and answer options of “1 day or less,” “2 days,” “3 days,” “4 days,” “more than 4 days,” “my milk never came in,” and “unsure/don’t remember”	Survey item adapted from 2 items validated with infant test-weight data [36]	PP wk. 2
*Perceived milk supply	Sum subscale score on Likert scale instrument measuring perceived infant satisfaction with breastfeeding/breast milk received; Checklist item in problem list (“not enough milk”) and forced choice item classifying perception of milk supply ("too much," "about the right amount", or "not enough")	PIBSS subscale of H & H Lactation Scale (5 item) [33]; investigator-created items	PP wks 2, 6, 12
Interim prenatal breastfeeding confidence	Five-point Likert scale assessing agreement with single item, “I am feeling confident and well-prepared to breastfeed after my baby is born.”	Investigator-created item	At conclusion of each prenatal study visit, after intervention delivery

*EHR* electronic health record, *PP* postpartum, *wk*.(s) week(s)

^a^Wording of some items adapted from original instrument to indicate that “breastfeeding” is inclusive of direct chest/breastfeeding as well as pumping/expressing milk; items in scale pertaining to direct breastfeeding excluded for participants who reported they were not engaged in direct breastfeeding

### Study population

Broadly, eligibility criteria for this study are based upon feasibility of data collection, conditions outside of the intervention expected to significantly impact milk volume and lactation outcomes, and circumstances expected to interfere with intervention delivery.

Inclusion criteria: (1) pre-pregnancy BMI ≥ 25 kg/m^2^ via electronic health record (EHR); (2) ≥ 18 years; (3) English-speaking (expansion to include Spanish-speaking populations is in-progress); (4) 34^0/7^–36^6/7^ gestational weeks; (5) nulliparous (i.e., no prior births at 20 or more gestational weeks); (6) intention to/interest in breastfeeding/provision of one’s own milk after birth; (7) singleton pregnancy; (8) plan to receive prenatal care and give birth at select birth centers/healthcare systems based in Pennsylvania; (9) own a phone with an unlimited text message plan; (10) access to technology allowing for video-based remote visits (e.g., cellphone with camera, internet).

Exclusion criteria: (1) contraindications to breastfeeding as specified by the American Academy of Pediatrics [37]; (2) history of breast reduction surgery or radiation (rationale: impact on milk production); (3) any prior history of induced lactation or breastfeeding; (4) indication for induction or birth by 37 weeks gestation as specified by American College of Obstetricians and Gynecologists (ACOG) (e.g., placenta previa, preeclampsia, small for gestational age fetus with abnormal dopplers; rationale: interference with ability to deliver intervention beginning at 37 weeks) [38]; (5) gestational or pre-existing diabetes (rationale: impact on lactation outcomes; research gap regarding efficacy of AME in a non-diabetic population).

### Setting

Eligible participants receive obstetric care at one of four health systems, which are based in Pittsburgh, Pennsylvania with additional study prenatal recruitment locations and birth hospitals/centers in north and central Pennsylvania. The primary birth center for the majority of participants is UPMC Magee-Womens Hospital (MWH), which is the Pittsburgh region’s largest obstetric hospital and referral center and a National Center of Excellence in Women’s Health. MWH has approximately 10,000 births  per year and its prenatal practices serve 11,000 new obstetric patients each per year. MWH accounts for 45% of all births in Allegheny County, where the population is 80% white, 13% Black/African American, 4% Asian, < 3% other races, and 2% Hispanic/Latinx [39]. The rate of breastfeeding initiation in 2018 was 81.2% in Allegheny County and 81.5% at MWH, both below the national average of 83.9% in the same year [40, 41]. On a broader scale, Pennsylvania in 2018 ranked among the lower half to lower one-third of U.S. states for breastfeeding initiation, exclusivity at 3 and 6 months, and continuation to 12 months postpartum [42].

None of the birth hospitals/centers in the study are designated as Baby-Friendly Hospital Initiative (BFHI) facilities. All study birth centers either have Keystone 10 designation or are on the pathway toward that designation. MWH is on the Keystone 10 pathway. The Keystone 10 Initiative is a program developed through the Pennsylvania Department of Health to assist Pennsylvania birthing facilities to improve breastfeeding support and rates. There are 10 steps to achieve designation, inclusive of having written lactation support policies and procedures, similar to the BFHI [43]. Prenatal and community/county-level lactation support programs within the regions in which study participants are recruited and reside vary widely.

### Recruitment

We currently employ a variety of remote and in-person participant recruitment strategies. We scan electronic health records (EHRs) of prenatal practices involved in the study for patients who are at least 28 weeks pregnant and meet basic eligibility criteria (parity, pre-pregnancy BMI, etc.); these patients are sent postcard study advertisements, and some receive a study brochure, messaging through their health system portal, or are approached by study staff at a prenatal visit. We also recruit through targeted social media ads and emails, a university research registry, paper and electronic study flyers at clinical sites, magazine advertisements, and advertising through social media of community organizations that serve pregnant individuals and families. For prospective participants with whom contact is established prior to 34 weeks of pregnancy, we offer preliminary eligibility screening and contact these individuals again between 34 and 36^6/7^ weeks of pregnancy for formal screening and enrollment. Screening and enrollment occur either in-person or remotely via a University HIPAA (Health Insurance Portability and Accountability Act)-secure Zoom platform. When remote, written informed consent is completed electronically. Participants are compensated with cash card at each data collection point—up to USD $320 total.

### Sample size

Our targeted enrollment sample size is 280 birthing parents. This sample size was based on an anticipated intervention drop-out/attrition by 2 weeks postpartum of 25%, current national breastfeeding exclusivity rates at 1–2 weeks postpartum, effects sizes for lactation support interventions in similar populations [19, 44], and anticipated challenges of recruitment within our eligiblity and geographical contraints. With a sample size of at least 210 (105 per group), we can detect with .80 power between-group differences in the prevalence of breastfeeding exclusivity at 2 weeks postpartum as small as .17 (medium effect size of OR = 2.55 using likelihood ratio chi-square test statistics) at an adjusted test-wise significance level of .017. In addition, with this sample size, we will be able to detect small to medium interaction effects between treatment groups over time as small as *f* = .32 when using repeated measures F-tests with at least four postpartum outcome time points.

### Randomization

After enrollment and prior to any data collection, participants are allocated to a group via opaque sealed-envelope, computer-generated permuted 2- and 4-block randomization, stratified by pre-pregnancy BMI status (BMI: 25–29.9 kg/m^2^ or BMI: ≥ 30 kg/m^2^). To maintain participant and clinical provider blinding to study purpose and decrease the likelihood of cross-contamination of treatments between groups, the oral presentation of the study and study materials describe the study purpose in broad terms as an investigation of the effect of antenatal video-based education on maternal postpartum experiences and behaviors. Potential group assignments are discussed without reference to primary lactation outcomes of interest.

### Intervention delivery

Participants in both groups meet with study personnel for remote video visits via HIPAA-compliant Zoom during week 37, 38, 39, and 40 of pregnancy to receive their assigned intervention if they are still pregnant and have not developed exclusion criteria. Visits are approximately 15–30 minutes, and participants use their personal devices (e.g., cell phone) to access the visit. Study personnel (and IBCLC interventionists for the AME group) conduct visits in separate remote private locations, and visits are electronically “locked” to maintain privacy. A description of the assigned intervention and risks and benefits are documented in participants’ electronic health records to alert providers to study participation.

### Experimental intervention: antenatal milk expression (AME) education

**Fig. 1:**
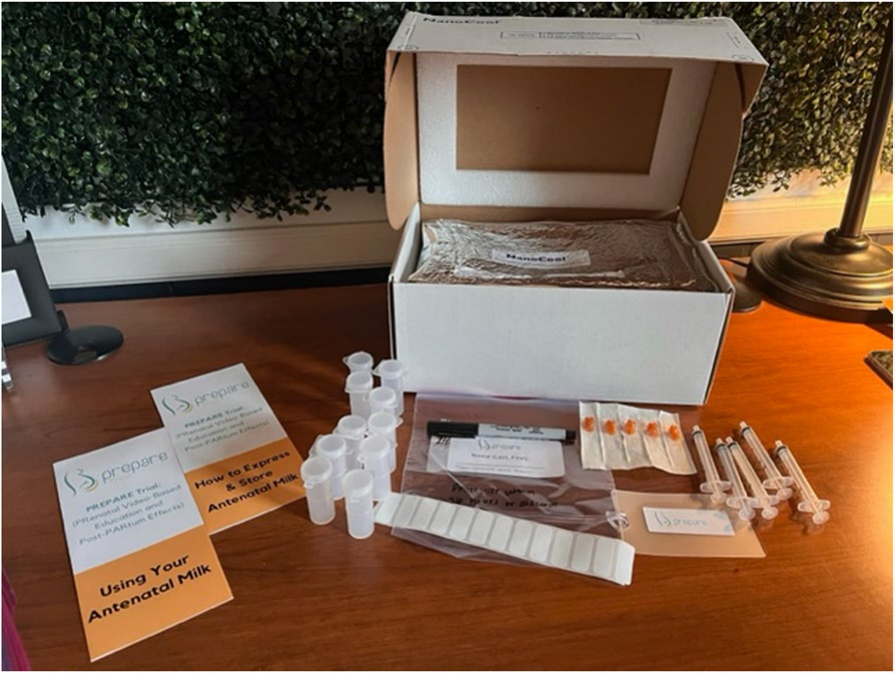
Antenatal milk collection kit. Kit includes milk cooler, milk collection containers (flip-tops and 3 mL syringes with caps; 1 mL and 5 mL syringe sizes not available at time of photo), plastic bag for milk containers at time of transfer to cooler (with sticker to note time of milk removal from home freezer), labeling tape, waterproof marker, study luggage tag, and brochures/instructions on antenatal milk expression and collection and use of antenatal milk

**Fig. 2:**
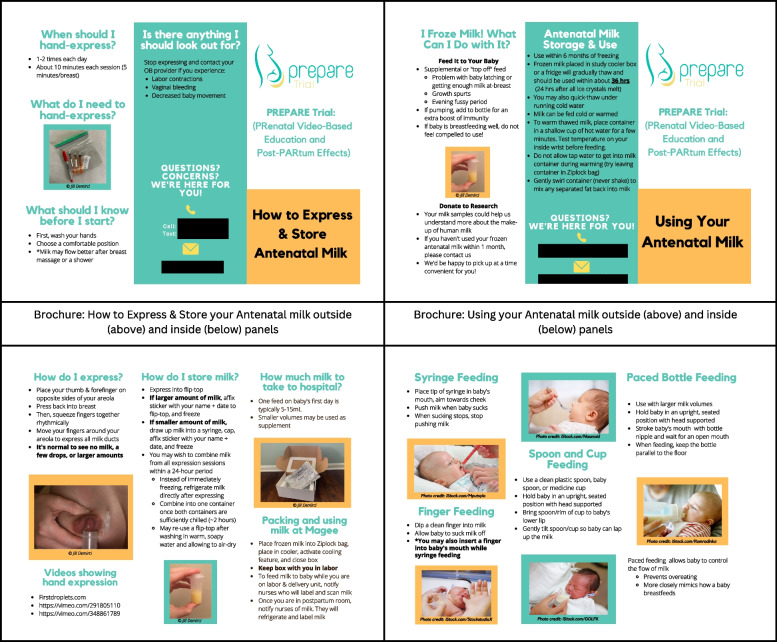
Instructional brochures in the antenatal milk collection kit. Brochures provide instructions on: 1) antenatal milk collection and storage; and 2) use/feeding of antenatal milk

After enrollment but prior to the first study visit, participants randomized to AME education are sent a milk collection kit and two brochures created by study personnel addressing how to hand express, collect, store, and feed any expressed milk (Figs. 1-2). The milk collection kit includes an insulated foam box with self-activated coolant in the lid for transportation of frozen antenatal milk to the birth center [45]. The kit also includes five 1-mL syringes, five 3-mL syringes, three 5-mL syringes, and ten 11 mL flip-top Snappies® colostrum collectors, labeling tape, a waterproof marker, and a luggage tag with study logo. Participants are instructed that the luggage tag can be affixed to their birthing/hospital bag as a reminder to bring any frozen antenatal milk to the birth center with labor onset (Fig. 2).

**Fig. 3:**
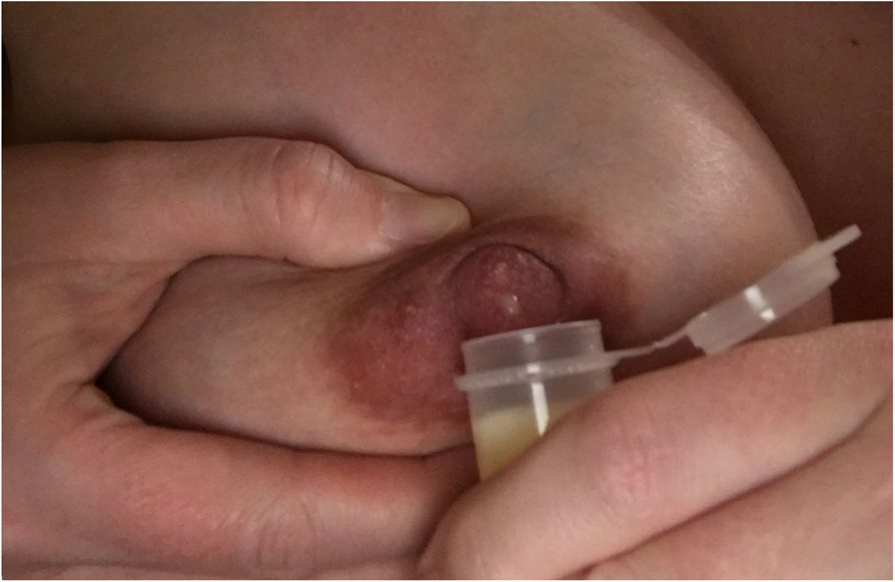
Example of antenatal milk expression and milk collection technique taught as part of the AME educational intervention. Note that photo is from the first author’s own photo collection. The person expressing is not a participant in the current trial

Participants are taught the milk expression technique during the 37-week Zoom visit after first viewing two introductory videos demonstrating the technique. The first video features an IBCLC teaching a pregnant person how to hand express milk with one hand using the Marmet technique [46]. Milk collection is demonstrated with a syringe. The second video demonstrates a postpartum individual hand-expressing using a two-handed technique [47]. Milk collection is demonstrated with a small open-mouthed collection container. Following the videos, a remote-based IBCLC from Pacify Health trained in the milk expression study protocol provides guided feedback on AME and milk collection as the participant engages in the technique for a maximum of 10 minutes (Fig. 3). IBCLCs are also encouraged by study staff during the call to address any questions on AME or general lactation questions. If the participant is amenable, study staff request that any milk collected during visits (if ≥1 mL) should be frozen in the participant’s home freezer and set aside for later pick-up by study staff for future analyses.

Participants are then instructed to practice AME 1–2 times per day for up to 10 minutes per session and store any expressed milk in provided containers labelled with their name and date and time of expression in their home freezer, in line with a prior published protocol for AME among diabetic pregnant women [19, 48]. Automated short message service (SMS, i.e., text message) queries using branching logic are then sent to participants’ cell phones daily until birth to assess frequency and duration of AME practice for the previous day, any problems experienced, and volume of milk collected.

At the subsequent three study visits during the 38th, 39th, and 40th week of pregnancy, participants meet with a Pacify Health IBCLC and study personnel to practice AME for a maximum of 10 minutes and receive reinforcement and feedback on their technique. At each visit, instructions for milk collection, storage, and transport to the birth center are reviewed, as well as indications for discontinuing AME (vaginal bleeding; regular/frequent labor contractions, reduction in fetal movement, development of exclusion criteria). While participants are encouraged to practice AME on camera to receive feedback from the IBCLC, for participants uncomfortable with breast exposure, study staff suggest turning off the camera or pointing the camera away from their breasts while the IBCLC attempts to have the participant describe what they are seeing and feeling to provide feedback.

### Comparison/attention-control condition: infant care education

**Fig. 4:**
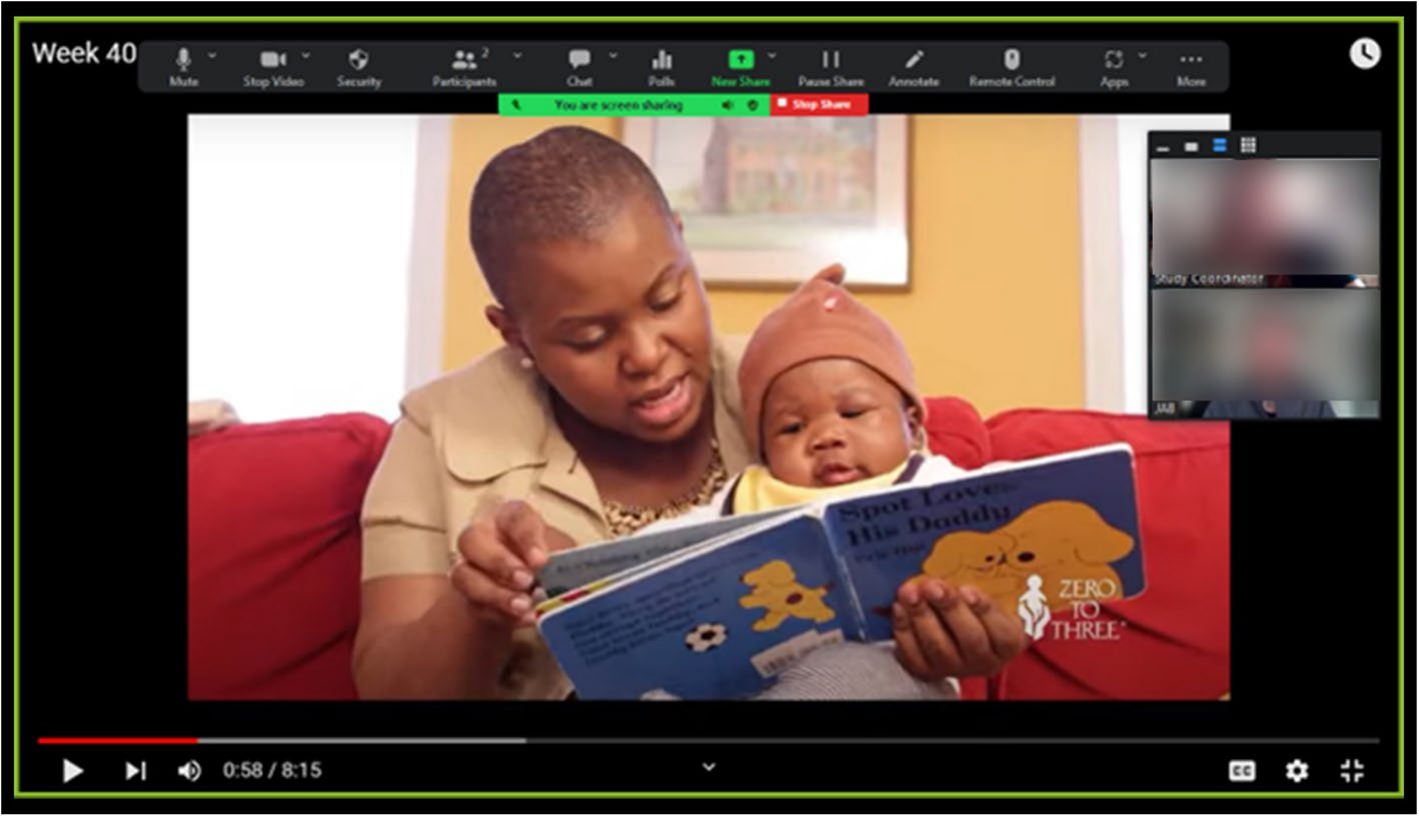
Screenshot from a video visit with a control group participant in the 40th week of pregnancy. Video featured addresses infant language and literacy development

To stem threats to internal validity resulting from unequal attention between groups or lack of treatment to incentivize continued study participation, video visits with control group participants occur at the same weekly frequency and in similar duration to the intervention group. During visits, study staff stream videos for participants addressing evidence-based infant care, all unrelated to infant feeding (Fig. 4). Videos focus on a different theme each week, including safe sleep, techniques to calm and soothe infants, car seat safety, and language and literacy development. Videos were vetted by two primary care pediatric providers and include selections from HealthyChildren.org via the American Academy of Pediatrics [49], Zero to Three [50], and content from independent health providers and health systems.

### Data collection

**Fig. 5:**
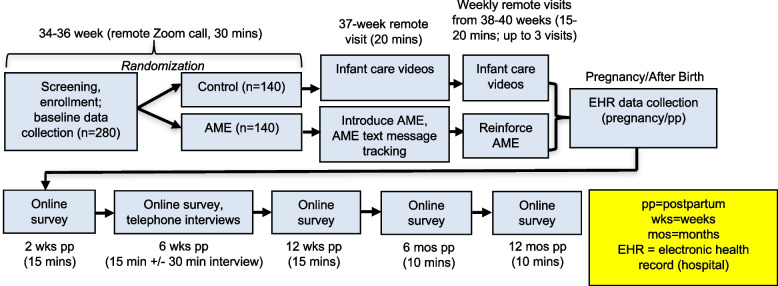
Study flow diagram

At each prenatal study visit, study staff query participants about any health changes and problems with AME since last visit (if applicable). At the conclusion of the study visit, participants are asked about their current confidence related to breastfeeding and those in the AME group are asked to provide an overall rating of the IBCLC interventionist. Participants complete survey measures at enrollment (34–36^6/7^ weeks of pregnancy) and postpartum at 2, 6, and 12 weeks and 6 and 12 months (Fig. 5). Study staff email surveys through the REDCap platform [51]. If no response to the emailed survey is received, study staff attempt to complete the survey by telephone. Data on participants’ pregnancies and postpartum birth center courses are collected from the EHR, including pre-pregnancy BMI and gestational weight gain, maternal and infant health conditions, labor and birth information, and infant feeding practices during birth center admission.

An individual not involved with study enrollment or visits who has qualitative interview expertise conducts individual telephone interviews with participants from both groups at 6 weeks postpartum or at the time of breastfeeding cessation if prior to 6 weeks. The goal of the interviews is to understand participants’ experiences with AME education and to improve study processes. Participants are selected purposively for interviews based on variability in intervention uptake, demographics, and infant feeding method to achieve a representative group. We estimate that approximately 25% of the study sample (*n* = 35 per group) will provide sufficient data to achieve saturation in themes/views, though more interviews may be conducted as study processes are modified and/or AME clinical recommendations or practices evolve. Interviews follow a semi-structured guide which broadly addresses motivating factors for study participation; perceived benefits/utility of AME and/or antenatal video-based infant education; experiences with other lactation and infant education support resources; experienced or anticipated challenges with AME and the control condition; and for AME participants, contextual factors impacting AME uptake and use of antenatal-expressed milk and suggested modifications to the AME intervention. Interviews are audio-recorded and transcribed verbatim using NVIVO cloud transcription. Transcripts are checked for accuracy against the audio recording by study staff.

#### Conceptual framework

Measured outcomes and covariates were informed by Breastfeeding Self-Efficacy Theory and Life Course Theory, both of which have been used as explanatory models of breastfeeding behavior [52–54]. Life Course Theory posits that health behaviors, in this case breastfeeding uptake and continuation, are influenced by a birthing person’s unique life trajectory, including the biological/genetic, economic, historical, cultural and political context they inhabit; family and social network resources; personal events; and intergenerational effects [54, 55]. These contextual variables are included as moderating variables in the study. Breastfeeding self-efficacy is a measure of one’s confidence in breastfeeding and is considered a modifiable factor influencing breastfeeding duration [56]. In this study, we conceptualized breastfeeding self-efficacy as a main outcome influenced by AME, as well as a mediating factor in the relationship between AME and breastfeeding exclusivity and duration. Timing of onset of lactogenesis II and perceived milk supply/volume are also considered secondary outcomes and potential mediating variables impacted by AME and influencing breastfeeding outcomes.

Outcomes and covariates are measured via: 1) survey items or instruments previously validated in populations of birthing people; or 2) investigator-created items. In the former, item wording was sometimes modified to reflect timing of assessment/recall period, various methods of feeding one’s own milk, and gender inclusive terminology. Investigator-created items were trialed and modified for brevity and comprehension in the first author’s prior research with pregnant and postpartum people (Tables 1 and 2).’

**Table 2:** Covariates/moderating variables and measurement

Variable	Measurement	Source	Assessment timing
Intervention receipt/dose	Number of AME study visits and home-AME sessions	Investigator study tracking system, SMS queries	Daily from 37 wk. visit to birth
Antenatal milk volume	Total volume of antenatal milk expressed during study and average volume per AME session, in milliliters	SMS queries	Daily/weekly from 37 wks to birth
Demographic characteristics	Age, race, ethnicity, gender, Special Supplemental Nutrition Program for Women, Infants, and Children (WIC), education, employment, relationship status, size of household	IFPS-II adapted and investigator-created items	Enrollment
Breastfeeding attitudes and intentions/plans	Sum scale score measuring extent of agreement with statements about breastfeeding attitudes on Likert scale; sum scale score measuring extent of agreement with statements about breastfeeding duration and exclusivity on Likert scale; anticipated infant age of formula/ other foods introduction and stopping breast milk feeds	Iowa Infant Feeding Attitude Scale (13 items) [57]; Infant Feeding Intentions Scale (3 items)^a^ [58]; IFPS-II adapted items	Enrollment
Prior breastfeeding exposures, inter-generational effects	Whether participant and partner were breastfed (yes/no), proportion of friends/relatives that breastfed, perceived importance of others’ feeding opinions on Likert-scale	IFPS-II adapted items	Enrollment
Birth characteristics and birth center course	Birth mode, anesthesia, neonatal intensive care unit admission, infant birthweight, length of birth center admittance	Birth center EHR abstraction	Birth center admittance
Participant and infant health status	Various chronic, pregnancy, birth associated morbidities checklist	Prenatal and birth center EHR abstraction, investigator-created survey items	Enrollment and birth center admittance
Participant weight	Pre-pregnancy body mass index, gestational weight gain	Prenatal and birth center EHR abstraction	Enrollment and birth center admittance
Anxiety and depression	Frequency of feelings or thoughts	Perceived Stress Scale (4 items) [59]; PRAMS anxiety-depression (3 items) [60]	Enrollment, PP wks 2, 6, 12; 6 & 12 mos
Breastfeeding practices in birth center and home and milk expression details	Timing of first direct breastfeed, average LATCH score at birth center [61], number of lactation consults, number of direct breastfeeds and expressed milk feeds 24 hours prior to birth center discharge; mode of own milk feeds, frequency and methods used to express and feed one’s own milk	Birth center EHR abstraction, IFPS-II adapted and investigator-created items	Birth center admittance and PP wks 2, 6, 12; 6 & 12 mos
Breastfeeding support and education	Checklist of support from various resources, workplace breastfeeding support checklist, extent of agreement about support quality	IFPS-II adapted and investigator-created items, Breastfeeding Friendly Worksites Scorecard adapted items [62]	Enrollment, PP wks 2, 6, 12
Breastfeeding problems	Checklists of breastfeeding problems, reasons for formula use, reasons for breastfeeding cessation	IFPS-II adapted items	PP wks 2, 6, 12; 6 & 12 mos
Breastfeeding satisfaction	Extent of agreement with statements about breastfeeding	MIBSS subscale of H&H Lactation Scale (5 items) [33]	PP wks 2, 6, 12
Embodied weight stigma	Subscale score of instrument assessing frequency of body image psychological discomfort, shame	Weight- and Body-Related Shame and Guilt Scale (WEB–SG), Shame subscale (6 items) [63]	Enrollment
Traumatic birth experiences	Sum scale of Likert-scale instrument assessing stress, fear, and partner support surrounding birth	Birth Experiences Questionnaire (10 items) [64]	2 wk
Racial discrimination	Sum scale of instrument assessing frequency of perceived racial discrimination experiences	Everyday Discrimination Scale (9-item) [65]	Enrollment

*EHR* electronic health record, *PP* postpartum, *wk*.(s) week(s)

^a^Only items 3–5 included from Infant Feeding Intentions Scale, as items 1–2 part of study eligibility (score range 0–12, with higher score indicative of stronger intention to exclusively breastfeed up to 6 months postpartum)

### Intervention Fidelity, blinding, and data monitoring

IBCLCs who provide AME education are trained on the study milk expression protocol at an initial Zoom session with the study team; they are also provided a video and written guide about the protocol. The study principal investigator (PI) observes a subset of study video visits regularly and provides oral and written feedback to individual IBCLCs as needed to ensure consistency in teaching AME technique.

In both intervention and control groups, a checklist is completed by study personnel concurrently with each visit to document completion of each part of the intervention, duration of the visit, any deviations from the visit/study protocol, any additional lactation education provided, and any technical problems encountered. The checklist is also completed by the PI during observed study visits and compared for consistency with the research staff’s form.

The study biostatistician will be blinded to group assignment when conducting quantitative analyses. IBCLCs delivering AME education are blinded to study purpose and outcomes of interest. Participants and obstetric providers in recruitment clinics are also blinded to study purpose.

To assess unplanned intervention cross-over, we query control group participants at the 2-week survey about whether they attempted any milk expression during pregnancy, source of information/where they learned AME, and whether milk was collected and/or fed to the infant. This assessment was added post-hoc after a portion of enrolled participants passed the 2-week assessment, so these questions are also included in the 12-month survey. Any participants who had previously completed their 2-week survey, but self-reported discontinuation of all breastfeeding before the 12-month survey were contacted to complete this question.

In terms of data monitoring, study personnel review all remotely-administered surveys for completeness and response inconsistencies; we attempt to contact study participants for clarification on such items. Student research assistants provide a second check of all data completed by study personnel, including abstraction of electronic health record data. The PI reviews data collected from a subset of participants each month for completeness and accuracy.

### Planned analyses

We will use SAS (v. 9.4 or later; SAS Institute Inc., 2022) for exploratory and screening data analyses, missing data estimation, repeated measures modeling, event time modeling and moderation analyses. Mplus (v.8; Muthen & Muthen, 2022) will be used for possible mediation analyses.

#### Quantitative analyses

To examine the efficacy of the remotely-delivered AME intervention relative to the attention control on short- and long-term breastfeeding outcomes, an intent-to-treat (ITT) approach will be used, where all participants will be included in analyses as randomized, regardless of protocol adherence/deviations, treatment received, or withdrawal. Sensitivity of the results using ITT will be explored to identify the effects of the amount of intervention received (e.g., number of AME study visits) and deviations in protocol (e.g., unplanned intervention cross-over) on outcomes.

Generalized linear mixed-effects modeling with linear contrasts will be used to examine the effect of treatment assignment (AME vs. attention control) for each repeatedly assessed lactation outcome, with treatment group assignment as the between-subjects factor, time as the within-subjects factor, and an interaction between time and treatment group. Random effects for participants will also be included. Fixed and/or time-dependent covariates (e.g., pre-pregnancy BMI category) may be included secondarily to adjust for group imbalances or variables related to the dependent variables based on the literature or data screening results. Standard fit criteria (e.g., AIC and BIC) also will be used to identify the best-fitting repeated measures covariance structure. F-tests will test the main and interaction effects included in the model. Individual regression parameters will be estimated with confidence intervals. Sensitivity analyses will be performed to discern the impact of influential cases on modeling results. Linear contrasts will be specified in repeated measures models to test whether the AME intervention demonstrates greater improvements in lactation outcomes versus the attention control at each time point, in particular, when conducting hypothesis testing on the primary short-term outcomes. For event history type outcomes (e.g., breastfeeding duration, days to the onset of lactogenesis II, days to any formula), Cox proportional hazards regression methods will be applied to allow for possible censoring of the event of interest and inclusion of the fixed and time-dependent predictors.

To explore possible moderators of the treatment efficacy of the AME intervention relative to the attention control, the generalized linear mixed-effects models for repeatedly assessed breastfeeding outcomes will be expanded to include the potentially moderating variable and its interactions with the other model terms (treatment group, time, treatment group by time). To explore possible mediation by the identified proximal/intervening variables (e.g., breastfeeding self-efficacy, onset of lactogenesis II), mediational models will be fitted using structural equation modeling.

#### Qualitative analysis

At least two coders trained in qualitative data analysis will independently and iteratively code the first four to five transcripts (line-by-line) for major and sub- content/themes according to recommendations by Miles and Huberman [66]. Coders will compare their coding decisions, resolve discrepancies by consensus, and consolidate codes into larger categories. The remaining transcripts will be selectively coded, with double-coding of approximately 30% of transcripts. Qualitative analysis software (NVIVO) will be used to facilitate data handling, coding, and thematic analysis. Analysis will proceed concurrently with data collection, such that the interview guide may be modified to reflect emergent themes. Qualitative data analysis techniques, such as narrative summaries, interview titling, and matrices, may be used to aid in organizing and presenting findings.

## Discussion

This study seeks to address the efficacy of telelactation-delivered AME education on short- and long-term lactation outcomes to 1 year postpartum among non-diabetic first-time birthing people with pre-pregnancy BMIs ≥25 kg/m2. Prior to study launch in 2019, we had planned for study personnel to recruit participants and conduct study visits in-person following prenatal visits (connecting with IBCLCs remotely). However, the COVID-19 pandemic necessitated that we redesign our procedures for a fully remote study. To date in our recruitment and data collection efforts, this remote redesign seems to have mixed effects. Recruitment has posed challenges, both in terms of pace and recruiting a racially- and socioeconomically- diverse sample. Being more dependent upon prospective participants to initiate contact and express interest in the study, rather than approaching them to assess interest, has likely favored recruitment of individuals who have more flexible time and employment and have not experienced harm or discrimination in healthcare or clinical research settings. Conducting enrollment and study visits through Zoom also favors those with reliable internet connectivity, technological literacy, and availability of privacy at home or in the work environment. Conversely, remote visits have allowed us to offer considerable flexibility in scheduling and provide a measure of participant autonomy, such that participants may choose to hand express with the camera off or with the camera pointed away from their chest if uncomfortable with bodily exposure.

An important consideration in our study will be dose/exposure to the intervention and whether there were sufficient opportunities to practice AME and collect and bank milk prior to birth. Birthing people with pre-pregnancy overweight or obesity are already at an elevated risk for pregnancy complications necessitating labor induction or cesarean section prior to term [6, 8]. In addition, the prevalence of scheduled labor inductions at 39 gestational weeks for uncomplicated pregnancies is also likely to be high in our sample, based on findings from the ARRIVE Trial [67] and subsequent clinical recommendations from the American College of Obstetricians and Gynecologists [68].

We anticipate several other factors that may limit enrollment, uptake of AME, and the effectiveness of the intervention. For example, participants may receive clinical counseling from providers that raises concerns about the safety of AME. To avoid this scenario, we provide introductory sessions on the study to clinical providers and staff at all enrollment locations. Another issue that may dilute any potential intervention effect is our reliance on participants or their support person(s) to self-transport any antenatal milk to the birth center at the time of the birth admission. Storage of antenatally-expressed milk at the study birth centers ahead of the birth admission is not currently permitted. We have attempted to mitigate participant’s forgetting their milk at home by providing a study keychain that can be affixed to a packed bag for the birth center as a visual reminder. However, this does not overcome the fact that many participants are not admitted to birth centers from home. To minimize the improper storage and transport of antenatal milk, we provide participants written instructions on a brochure and the refrigerated milk transport box, oral instructions and reminders at each study visit, and a 24-hour per day number to call if any questions arise.

This study will be unable or underpowered to answer several important questions about AME. This includes optimal frequency and delivery method of AME education. In addition, the safety and risk/benefit ratio of beginning AME earlier than 36–37 weeks of gestation remains an open question, as does the nutritional and immunological comparability of antenatally-expressed and postpartum milk. It is also possible that our study is underpowered to detect small effects of AME on our main outcomes of breastfeeding exclusivity and breastfeeding self-efficacy at 2 weeks postpartum. By collecting additional data on breastfeeding outcomes at birth and over the postpartum period as well as qualitative participant experiences, however, we will be able to triangulate our data to assess AME’s value, feasibility, and efficacy in this specific population.

Conversely, this study will provide important insight into the value, safety, and feasibility of integrating AME into prenatal education in the United States and among understudied sub-groups at risk for early breastfeeding discontinuation (first-time parents, pre-pregnancy BMI ≥25 kg/m^2^). We will generate novel data for both AME’s effect on proximal lactation outcomes (e.g., breastfeeding self-efficacy), as well as more distal lactation outcomes (e.g., breastfeeding continuation). Our findings will add to the existing literature on the safety and side effect profile of AME to inform clinical recommendations. Finally, by collecting detailed data on participants’ experiences with intervention delivery, our study will provide insight on whether synchronous online video sessions are a desirable and feasible platform for AME education.

## Data Availability

Not applicable.

## Abbreviations

ACOG: American College of Obstetricians and Gynecologists
AME: Antenatal milk expression
BFHI: Baby-Friendly Hospital Initiative
BMI: Body mass index
EHR: Electronic health record
HIPAA: Health Insurance Portability and Accountability Act
IBCLC: International Board-Certified Lactation Consultants
ITT: Intent-to-treat
MWH: Magee-Womens Hospital of UPMC
NICU: Neonatal intensive care unit
PI: Principal investigator
PP: Postpartum
PREPARE Trial: PRenatal Video-Based Education and Post-PARtum Effects Trial
SMS: Short message service
